# Supplementary material for: Metastable hybridization-based DNA information storage to allow rapid and permanent erasure
Source: Nat Commun. 2020 Oct 6;11:5008. doi: 10.1038/s41467-020-18842-6 (PMC7538566; doi:10.1038/s41467-020-18842-6)
Supplement: Supplementary file 1 — Supplementary Information [file 41467_2020_18842_MOESM1_ESM.pdf]

Supplementary Information

Metastable Hybridization-based DNA Information Storage to Allow Rapid and Permanent Erasure

Kim *et al.*

Supplementary Notes 1-6	1-3
Supplementary References	4
Supplementary Figures 1-21	5-24
Supplementary Tables	25
Supplementary Data 1	attached

Supplementary Notes

Section 1: Truth Marker Binding Thermodynamics and Kinetics

The information solution is formulated by mixing the truth marker T with the correct message C and the false marker F with the noise message N. The TC + FN solution is metastable, because at equilibrium, all four species TC, FN, TN, and FC should be present at ratiometric concentrations, assuming that the binding of T to C and N are equally strong, and that the binding of F to C and N are equally strong. Thus, the hybridization states each message C or N to T or F encodes an orthogonal bit of information in addition to the information encoded by the sequences of C and N.

The binding of T to C is impermanent; given sufficient time, the metastable information solution will relax to the equilibrium state. We believe that there are two primary mechanisms for T to swap binding partners, illustrated in Supplementary Fig. 3 and Supplementary Fig. 4. The first mechanism is a unimolecular mechanism, through which TC and FN spontaneously dissociate into their single-stranded forms T, C, F, and N, which then randomly and ratiometrically form TC+FN or TN+FC. The second mechanism is a bimolecular mechanism, through which TC and FN bind to form an unstable intermediate TCFN, which can relax to either the TC+FN states or the TN+FC states.

The unimolecular mechanism is well understood and modeled, and is primarily rate-limited by the dissociation constant k_d of the TC and FN molecules. The value of k_{hyb} is assumed to be 10^6 M^{-1} s^{-1}, and the value of k_d is calculated based on $k_d = \frac{k_{\mathrm{hyb}}}{e^{-\Delta G^\circ/R\tau}}$, where $\tau = 273.15 + 30$ is the temperature in Kelvin, R is the ideal gas constant (Boltzmann constant), and ΔG° is the standard free energy of formation of the duplex formed by T and the truth marker binding region B. The value of ΔG° is calculated based on the sequence of T (see Supplementary Excel spreadsheet) and published DNA thermodynamics parameters. Summaries of ordinary differential equation simulations of the unimolecular mechanism are shown in Supplementary Fig. 3, based on experimental DNA oligo concentrations. As expected and consistent with experimental observations, the simulations show that the half-life of the information solution is strongly temperature-dependent, with more than 8 orders of magnitude difference based on a 20 °C change in storage temperature from 30 °C to 50 °C.

The bimolecular mechanism, in contrast, has not been systematically studied and modeled. A simple model of the mechanism is illustrated in Supplementary Fig. 4, but there are neither good characterizations of the k_{sd} or the k_{resolve} rate constants. The only experimentally confirmed information we have regarding k_{sd} is that it is slower than 1 M^{-1} s^{-1} [2–4] at 25 °C. Like most other chemical reaction rate constants, we expect that k_{sd} is temperature-dependent, with lower values at lower tepmeratures. Supplementary Fig. 4cd shows simulation results assuming $k_{\mathrm{sd}} = 0.1$ and 0.01 M^{-1} s^{-1}. From our experimental results in manuscript Fig. 4 and 5, we infer that the $k_{\mathrm{sd}} \leq 0.1$ M^{-1} s^{-1} at 25 °C.

Section 2. Gel Demonstration of Information Solution Storage and Erasure

We first used polyacrylamide gel electrophoresis (PAGE) to demonstrate the stability of the information solution at low temperatures and the rapid erasure of the information solution at elevated temperatures. For this experiment, we used just a single correct message C and a single noise message N, to facilitate unambiguous PAGE analysis. Furthermore, unlike our later experiments, we intentionally had the T oligo be 110 nt and the N oligo be 140 nt long, in order to facilitate distinguishing these two species in PAGE. Finally, unlike later experiments, the truth marker T was labeled at the 5′ end with a FAM fluorophore, and the false marker F was labeled at the 5′ end with a ROX fluorophore.

The PAGE experiments used an Invitrogen Novex pre-cast 10% acrylamide TBE gel with 12 wells (Catalog# EC62752BOX). The gel was run for 80 minutes at 100 V, at 25 °C in 1x TBE running buffer. The gel was scanned using a Typhoon FLA 9500 (G/E Healthcare) using 500V on the photomultiplier tube, and a resolution of 50 μm.

The same PAGE gel was scanned in the FAM and ROX channels before intercalating dye staining (Supplementary Fig. 5bc), and also after staining with the SybrGold dye. The results are generally consistent with expectations; there is not significant formation of TN and FC in lane 7 or 8, but they are present in lanes 9, 10, and 11. Because the concentrations of the TC and FN species are much higher in this experiment than in later experiments on Twist oligo pools encoding artwork images, we expect that the bimolecular pathway for truth marker binding randomization would be more significant here than in later experiments. The fact that we do not observe visible FC or TN formation in 1 week at 25 °C thus suggests that long-term information solution storage is achievable with our metastable encoding strategy.

Section 3. Information Encoding and Decoding Methods

DNA Data Encoding. For ease of visualizing information storage fidelity, we decided to encode images as bitmap files. In contrast, other image file formats such as JPG and PNG contain lossless or lossy compression, so that corruption of even a small minority of bytes/bits may render the image unreadable. In a standard 24-bit bitmap image file, each pixel corresponds to 3 bytes of information, corresponding to the Red, Green, and Blue intensities on a scale of 0 to 255.

Our information encoding scheme is illustrated in Supplementary Fig. 6. Five (5) nucleotides are needed to encode each byte, so 15 nt is needed to encode each pixel color. Given the synthesis limitations of Twist, the lengths of library primers, and the length of the truth marker binding site, we have roughly 70 nt per DNA oligonucleotide for the address and the message. This corresponds well to a 2-byte address (10 nt) and a block of 4 pixels (60 nt). Consequently, we organize each bitmap image into $\leq 255x255$ blocks of 2x2 pixels. For the pixel block corresponding to the four pixels with coordinates (99, 99), (99, 100), (100, 99), and (100, 100), we would encode both the X and the Y as 50.

DNA Data Decoding. Decoding the NGS reads back into the bitmap image starts through a process of filtering out reads with incorrect message lengths (Supplementary Fig. 7). These reads may include DNA oligonucleotide synthesis errors with internal deletions that could result in frameshift errors with many incorrect decoded bytes, and thus were judged to be not worth salvaging. As described in manuscript Fig. 3E, roughly 16% of all NGS reads were discarded. Of the remaining reads, we grouped reads by address, and proceeded with decoding the plurality sequence for each address.

Section 4. Oligonucleotide Pool, Sub-pool Amplification, and Spike-In Pool

The 8 bitmap images are encoded in a total 93,894 oligonucleotides, which were synthesized as a single pool by Twist Bioscience. Before using the oligos, we first ran NGS on the Twist oligos, with one NGS library corresponding to each image subpool. The protocol for NGS library preparation for these pools is different than from subsequent image reading and recording processes; see Methods. Supplementary Fig. 9 and Supplementary Fig. 10 show the sequencing results of the Twist libraries after amplification. A total of 784 pixels corresponding to 196 pixel block oligonucleotides (0.21%) were entirely missing, and 1 pixel was consistently wrong indicating synthesis error at one specific position. Additionally, another 778 pixel block oligonucleotides were present in the NGS library at very low coverage (<10x) relative to the median ($\approx$50x). To overcome the observed problems above, we designed a second Twist pool, known as the repair pool, with 975 oligos. The second pool was mixed with the first pool at a nominal 2:1 stoichiometric ratio per oligo. Supplementary Fig. 11 and Supplementary Fig. 12 show the sequencing results of the two combined Twist libraries; a total of 12 pixels (3 pixel block oligonucleotides) remain missing, indicating that roughly 1.5% of the missing oligos (3 / 196) are still missing in the repair pool.

Section 5. Efficiency of Information Solution Storage and Erasure

We first demonstrate information storage and erasure on a simple mixture of 2 files, corresponding to images 7 and 8, which have relatively similar dimensions (Supplementary Fig. 13). Storing the two separate information solutions (one with Image 7 being true and the other with Image 8 being true) for 1 week does not result in significant randomization of truth marker binding. In Supplementary Fig. 13a middle and 13c middle, no red pixels corresponding to incorrect pixel sequence are observed. After erasing via incubating at 95 °C for 5 minutes, the two erased information solutions closely resemble each other (Supplementary Fig. 13b and 13d). Note that the erased images look like a mixture of Images 7 and 8 because for every single pixel block, small biases in concentrations or PCR efficiencies of one of the two oligos with the same address become the plurality. Consequently, the middle images in Supplementary Fig. 13b and 13d appear to be negatives of each other.

We next demonstrate information storage using all 8 images, with 1 image pre-hybridized to the truth marker and the other 7 images pre-hybridized to the false marker. Eight separate information solutions with a different image pre-hybridized to the truth marker were created, and stored at 25 °C for different periods of time ranging 3 orders of magnitude, from 1 hour to 65 days (Supplementary Fig. 14, Supplementary Fig. 15, and Supplementary Fig. 16). Here, unlike with previous analysis on the information solution containing only 1 true image and 1 false image, the fraction of the NGS reads at each pixel that corresponded to the plurality may not be necessarily about 50%. From statistical analyses, there did not appear to be any significant difference in the number or fraction of pixels correctly recovered for different storage times.

Supplementary Fig. 17 and Supplementary Fig. 18 show the results of the 8 information solutions after 1 hour of

storage and subject to erasure via heating to 95 °C or 60 °C for 5 minutes. All 8 images in Supplementary Fig. 17 were statistically identical, but the 8 images in Supplementary Fig. 18 showed statistical differences in having higher than expected number of pixels matching the original image pre-hybridized to the truth marker. Thus, 5 minutes at 95 °C is sufficient for complete erasure, but 5 minutes at 60 °C does not fully take the information solutions to equilibrium.

Statistical Analysis of Erasure. To rigorously show that the DNA solutions erased via heating at 95 °C for 5 minutes (manuscript Fig. 4b and 5c), we examined the fraction of pixel blocks matched to each original image (Supplementary Table 1). It is clear that the Match Image with the highest fraction of matched pixel blocks is always Image 7, indicating that the biases in truth marker binding and NGS within each library dominate over differences across different libraries due to the original image information (Supplementary Fig. 19, left). Thus, for each library corresponding to each original image, only library 7 corresponding to original image 7 shows a plurality of correct pixel blocks.

We next asked whether, given the relative fraction of pixel blocks that match each Match Image, whether the original Image could statistically identified. In other words, does there exist a small but statistically significant increase in the number of pixel blocks that correspond to Match Image 1, for the library derived from Original Image 1? This analysis is plotted (Supplementary Fig. 19, right) and the p-values for each Match Image frequency was calculated using a one-way t-test. All 8 p-values were not statistically significant at the $\alpha = 0.05$ level.

We note that bulk statistical identification of which of the original 8 images each library corresponds to is a much lower bar than decoding the actual message, because we are determining 1 image out of 8 possible choices, rather than reconstructing 1 message from $8^{20,000}$ possible messages. Thus, we are highly confident that information restoration after erasure (5 min at 95 °C) is not possible.

As an alternative method of analyzing the maximum possible information that remains in the erased images, we computed the p-values for one-way t-tests on the null hypothesis that $\mathrm{M}(i,i) \geq \mathrm{Mean}(\mathrm{M}(i,j))$ + Delta, where $\mathrm{M}(i,k)$ corresponds to the fraction of matched pixels in row i and column k in Supplementary Table 1. Delta is the hypothesized maximum information that remains, for example the calculation for Delta = 0.05 evaluates the likelihood that M(1, 1) has a true value at least 0.05 higher than the mean of M(1, j), with $j \neq 1$, implying that 5% of the original information has been retained. The computed p values for different values of Delta are plotted for all 8 images in Supplementary Fig. 20. The p values for all 8 images are below $\alpha = 0.05$ when Delta ≥ 0.02, indicating that no more than 2% of the original information remains in the erased images.

Note that the Delta values implied by fixed p cutoffs represent an upper bound on the mean amount of information retained. As seen in Supplementary Fig. 19, several of the Erased Images actually have smaller fraction of pixels matching to their original image than Erase Images derived from other original images, suggesting that confident identification of the original image is not possible for these particular Erased Images.

Section 6. DNA Degradation by Other Methods

DNA is a relatively stable molecule, but many methods for intentionally destroying DNA have been presented, which can be thought of as methods for erasing information classically encoded in DNA. Here, we experimentally evaluated two methods for DNA destruction: UV irradiation at 254 nm and DNAse I digestion. We quantitated the degree of DNA destruction using qPCR for an amplicon 77 nt. Because incomplete degradation of DNA to below 77 nt in length would appear as degradation even though information may not be completely erased, the observed degradation inferred from Ct value is considered an **upper bound** on the actual degradation yield, rather than a true value.

Our results indicate that UV irradiation for 2 hours is not sufficient to complete destroy the DNA, but that 20 minutes of reaction with DNAse I at 37 ° in optimized buffer is sufficient. We note that DNAse I is a fragile enzyme with a limited shelf life that must be stored at -20 ° until use, and that the degradation by DNAse I required an initial labor-intensive buffer exchange step.

Supplementary References

[1] J. SantaLucia, D. Hicks, The thermodynamics of DNA structural motifs. *Annu. Rev. Biochem.* **22**, 415 (2004).
[2] B. Yurke, B., A. P. Mills, Using DNA to power nanostructures. *Genetic Programming and Evolvable Machines,* **4(2),** 111-122 (2003).
[3] D. Y. Zhang, E. Winfree, Control of DNA strand displacement kinetics using toehold exchange. *J. Am. Chem. Soc.* **131**, 17303 (2009).
[4] S. X. Chen, D. Y. Zhang, and G. Seelig, Conditionally fluorescent molecular probes for detecting single base changes in double-stranded DNA. *Nature chemistry,* **5(9),** 782 (2013).

Supplementary Figures

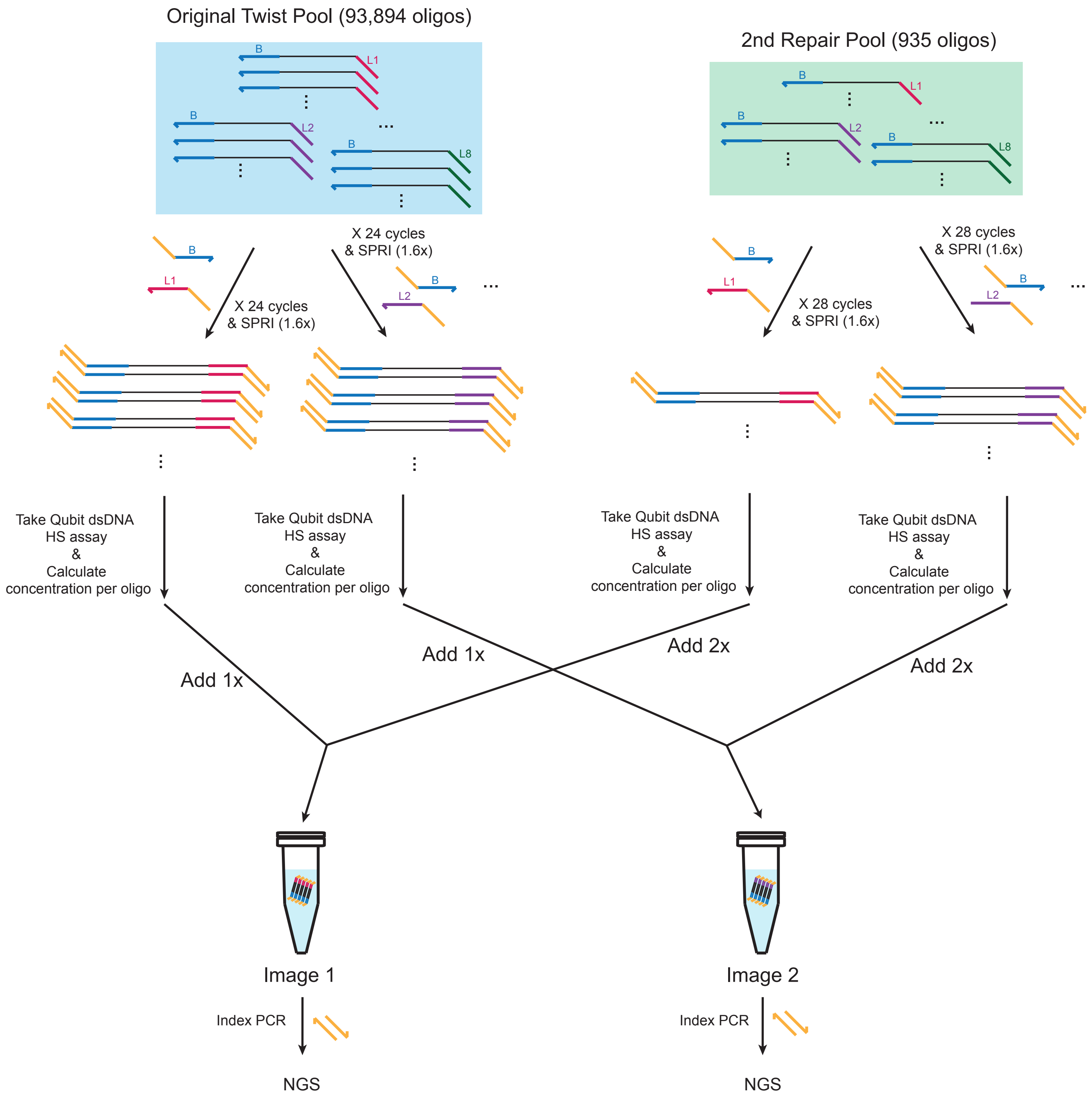

Supplementary Figure. 1: Twist oligo pool amplification workflow.

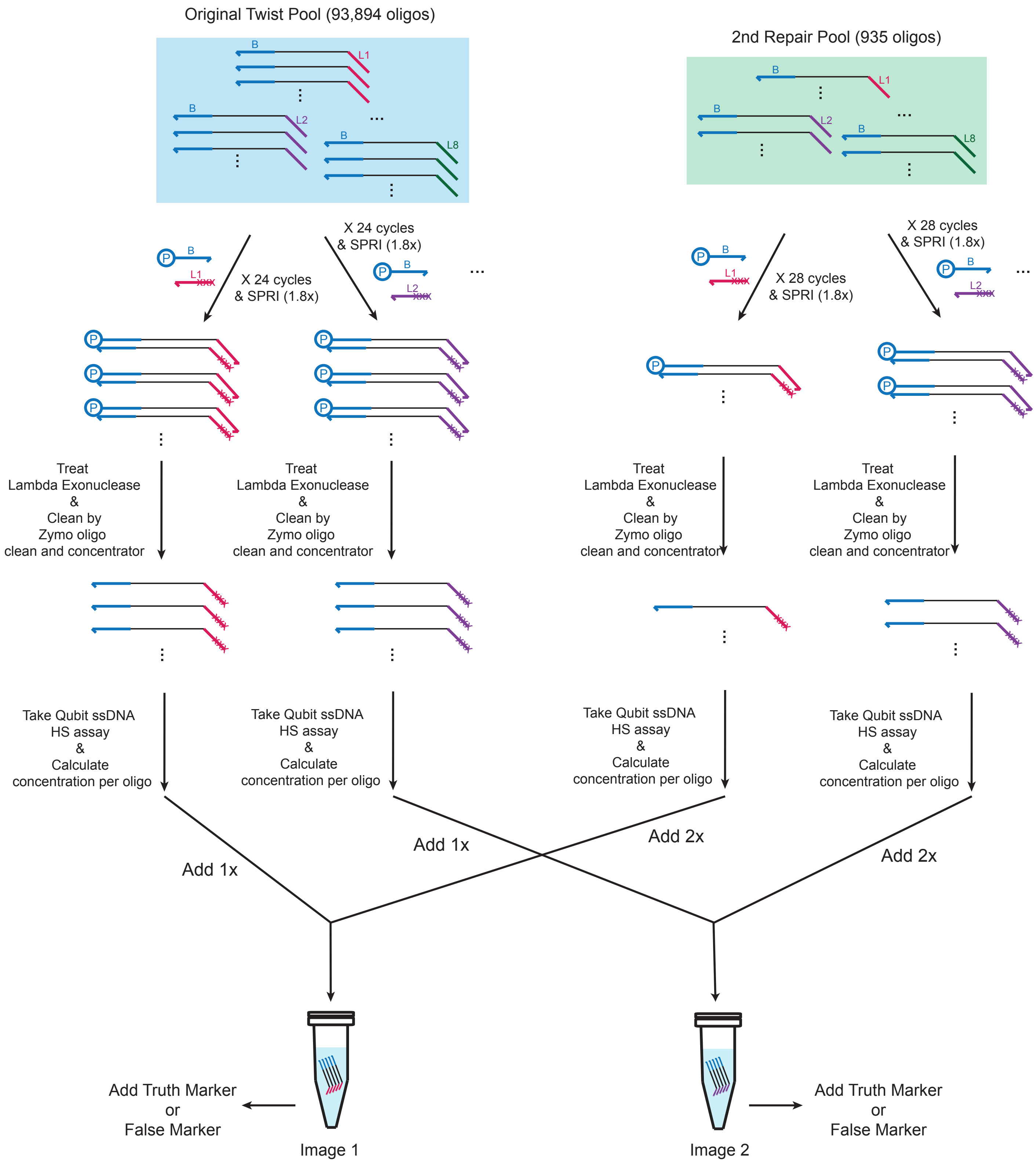

Supplementary Figure. 2: Workflow for formulating an information solution.

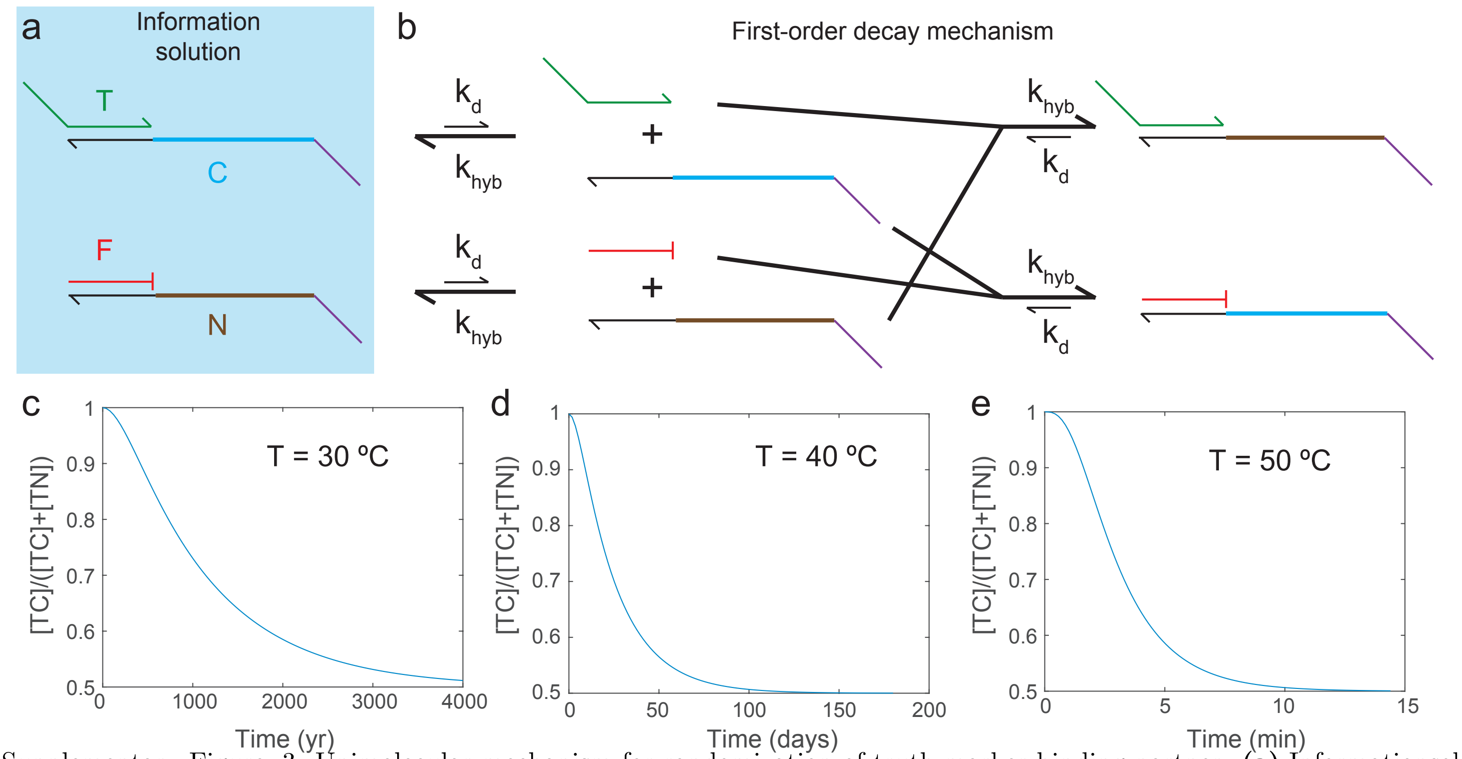

Supplementary Figure. 3: Unimolecular mechanism for randomization of truth marker binding partner. **(a)** Informationsolution comprising truth marker T initially bound to C and false marker F initially bound to N. **(b)** Spontaneous unimoleculardissociation of TC and FN produces free T, C, F, and N. The free T, C, F, and N bind to each other randomly, forming either TC + FN or TN + FC. **(c)** Ordinary differential equation (ODE) simulation results for information solution stability at 30 °C. The simulation assumes 1 false image at equal concentration to the intended image; thus, at $\frac{[TC]}{[TC]+[TN]} = 0.5$, the truth marker pairing has completely randomized, and no recovery is possible. The information half-life is roughly 1000 years (for $\frac{[TC]}{[TC]+[TN]}$ to reach 0.75). **(d)** ODE simulation results for information solution stability at 40 °C; the information half-life is roughly 20 days. **(e)** ODE simulation results for information solution stability at 50 °C; the information half-life is roughly 2 minutes.

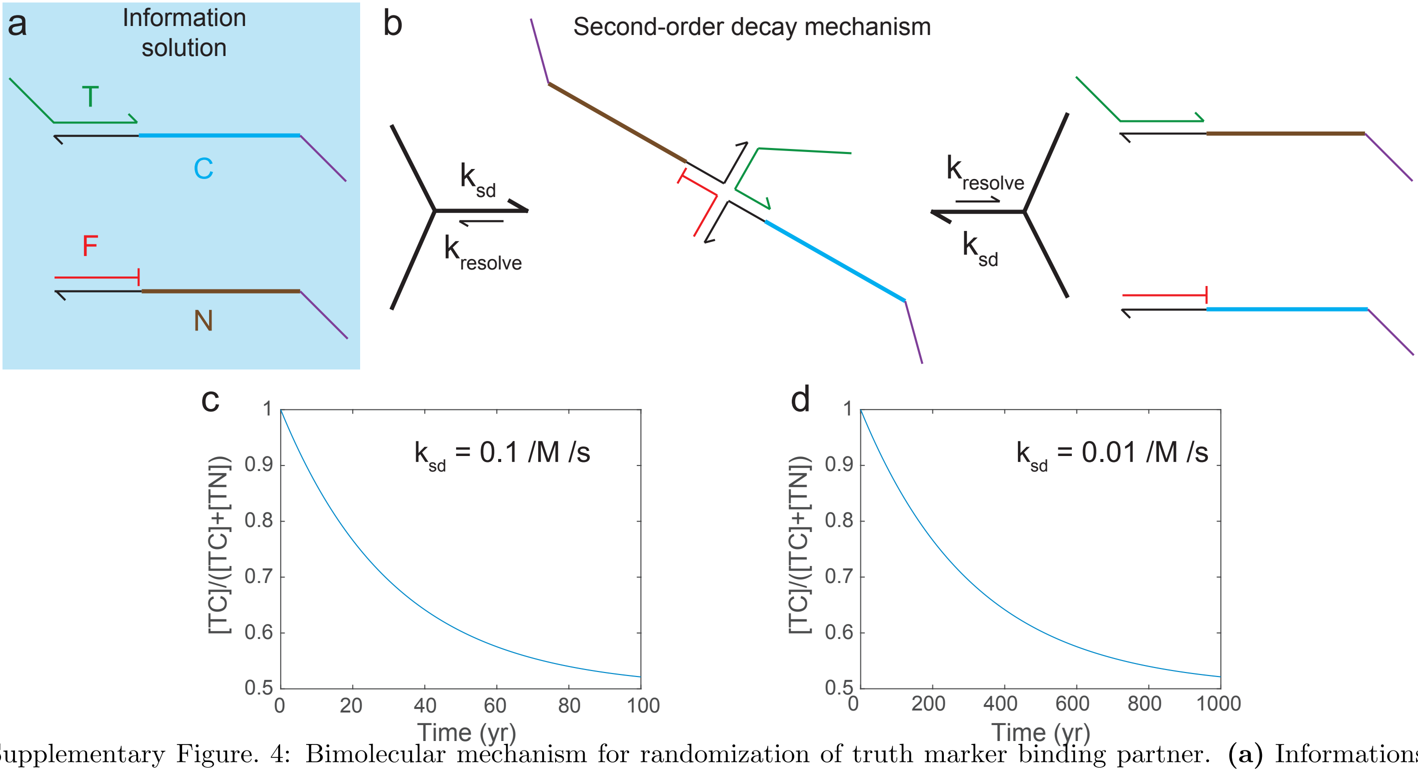

Supplementary Figure. 4: Bimolecular mechanism for randomization of truth marker binding partner. **(a)** Informationsolutio n comprising truth marker T initially bound to C and false marker F initially bound to N. **(b)** Bimolecular associationof TC and FN produces a transient 4-stranded complex TCFN, which can resolve into TC + FN or TN + FC. **(c)** Ordinary differential equation (ODE) simulation results for information solution stability, assuming $k_{\rm sd} = 0.1$ M^{-1} s^{-1}. The temperature dependence of $k_{\rm sd}$ is not well studied, so we do not specify the temperature here. Kinetics studies [**? ? ?**] suggest that $k_{\rm sd}$ should be no higher than 0.1 M^{-1} s^{-1} at room temperature (25 °C). The information half-life is roughly 20 years (for $\frac{[TC]}{[TC]+[TN]}$ to reach 0.75). **(d)** ODE simulation results for information solution stability assuming $k_{\rm sd} = 0.01$ M^{-1} s^{-1}; the information half-life is roughly 200 years.

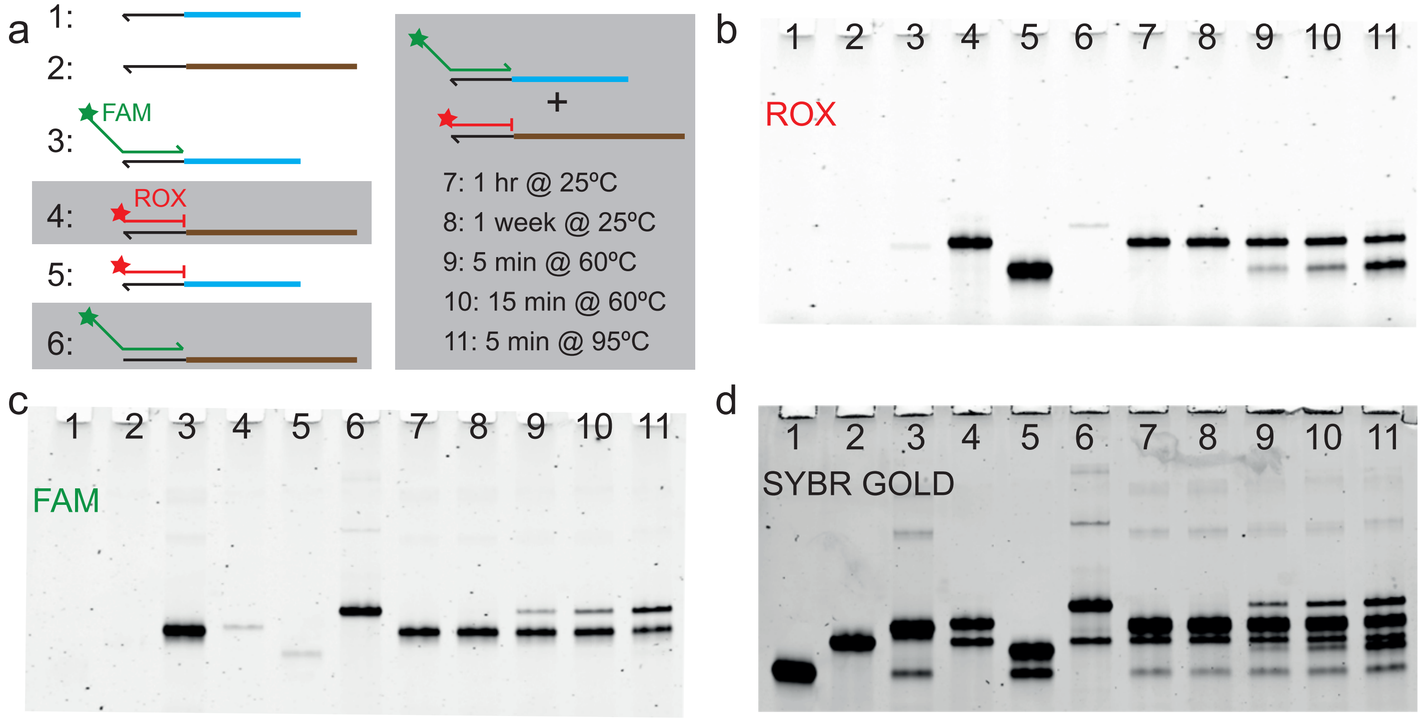

Supplementary Figure. 5: Experimental demonstration of truth marker binding stability via polyacrylamide gel electrophoresis. Manuscript Fig. 2c shows a condensed version of this figure, overlaying the ROX and FAM channel images and removing lane s1 and 2 for brevity. **(a)** Gel lane descriptions. Lanes 1 and 2 show the intended message and the noise DNA, respectivel y.Lanes 3 and 4 show the intended message pre-hybridized to the truth marker and the noise DNA pre-hybridized to t hefalse marker, respectively. Lanes 5 and 6 show the intended message pre-hybridized to the false marker and the noise D NApre-hybridized to the truth marker. Lanes 7-11 show the mixture of the species in Lanes 1 and 2 incubated for differ entamounts of time at different temperatures. **(b)** ROX channel image of the gel. The upper band corresponds to the FN spec ies,and the lower band corresponds to the FC species. **(c)** FAM channel image of the gel. The upper band corresponds to theTN species, and the lower band corresponds to the TC species. **(d)** Gel image after staining with SybrGold, showing allobserved DNA species.

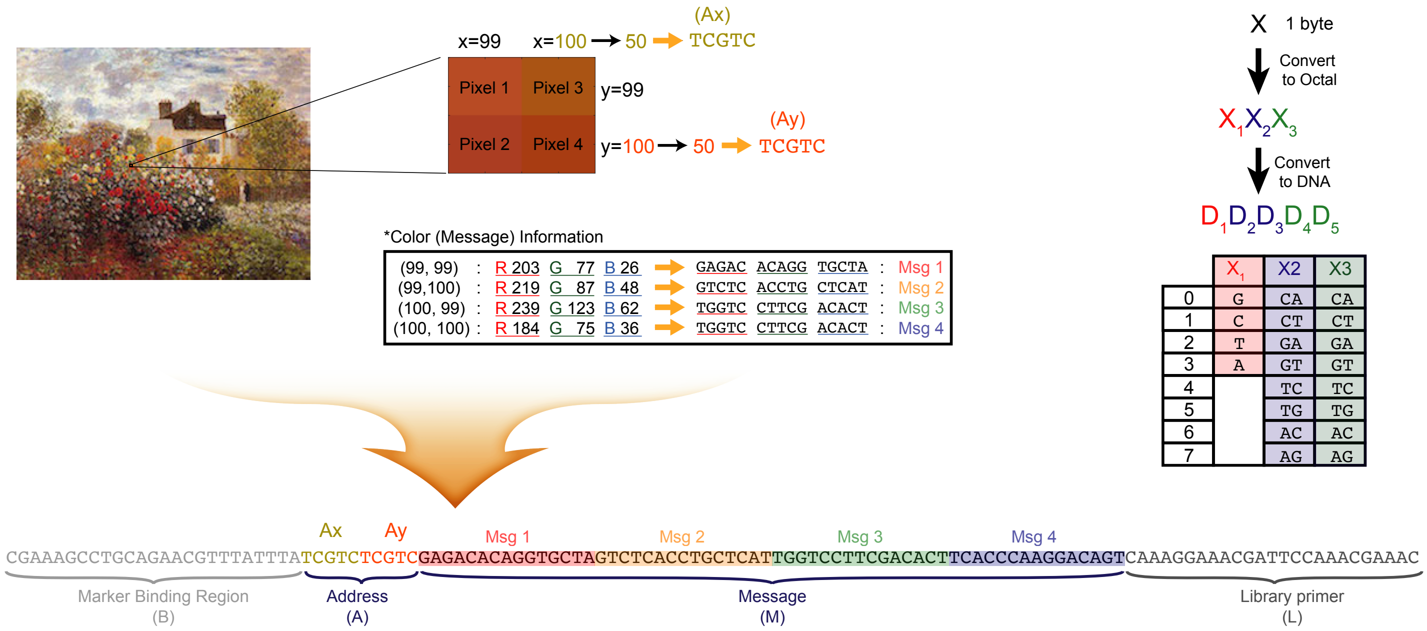

Supplementary Figure. 6: Encoding a bitmap image in DNA sequence. Each DNA oligonucleotide contains a 10 nt address Ac corresponding to a 2x2 pixel block, and a 60 nt message M corresponding to the RGB values for 4 pixels, in addition to the marker binding region B that is identical for all oligos and the library primer L that is identical for all oligos within this file .

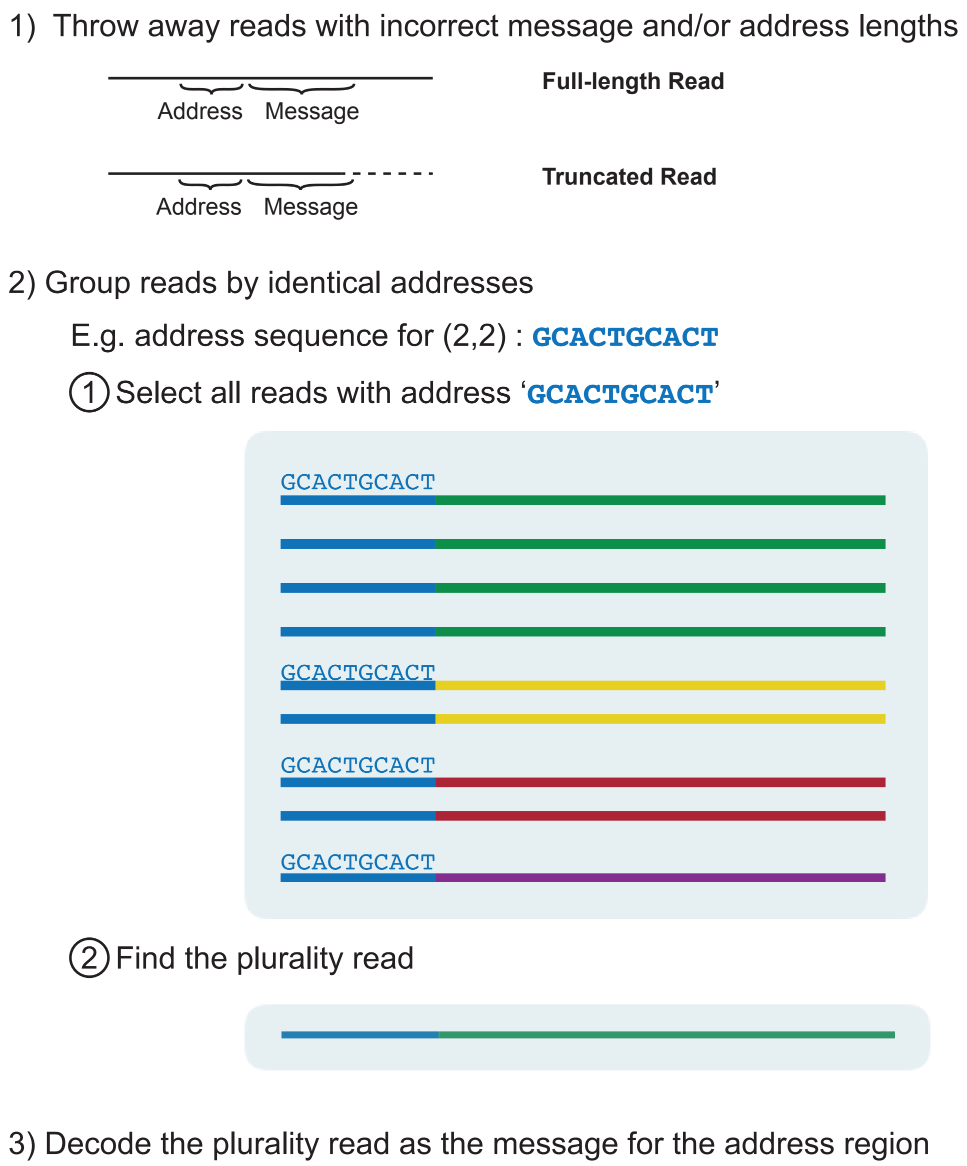

Supplementary Figure. 7: NGS read filtering. We remove all NGS reads in which the address/message sequence is not the exact expected length. The remaining NGS reads are grouped by address, and for each address the sequence with plurality o fNGS reads is selected as the sequence for further decoding.

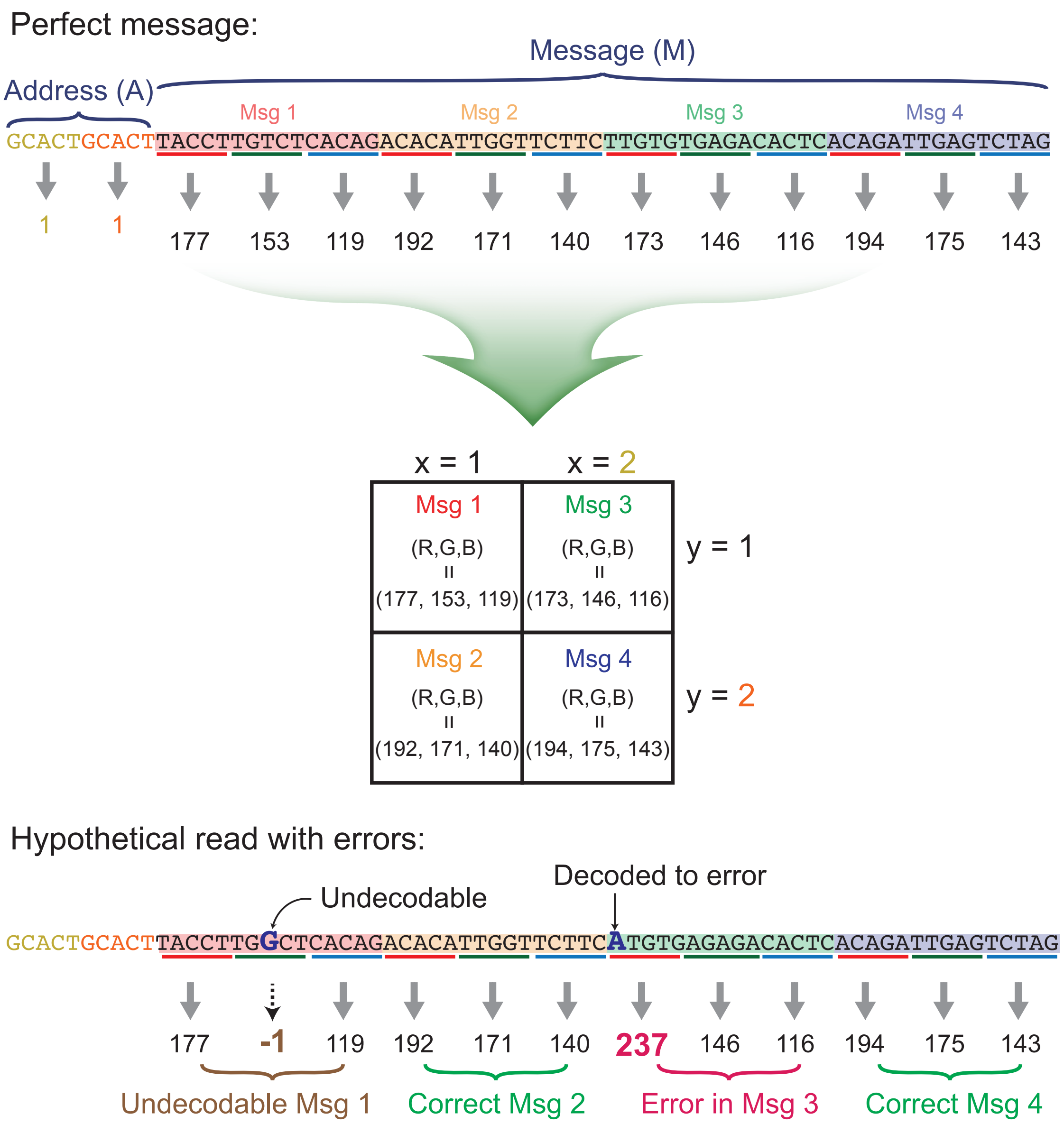

Supplementary Figure. 8: Decoding a NGS reads from FASTQ file into messages for reconstruction of the bitmap image. There two possible decoding error modes: (1) undecodable bytes that cannot be mapped back to a valid byte value between 0 and 255, and (2) incorrectly decoded bytes in which a synthesis or sequencing error read results in the byte value being decoded to a value other than intended. Each byte of the NGS read is decoded separately, so that the impact of occasional DNA synthesis errors is compartmentalized to a single R/G/B value of a single pixel.

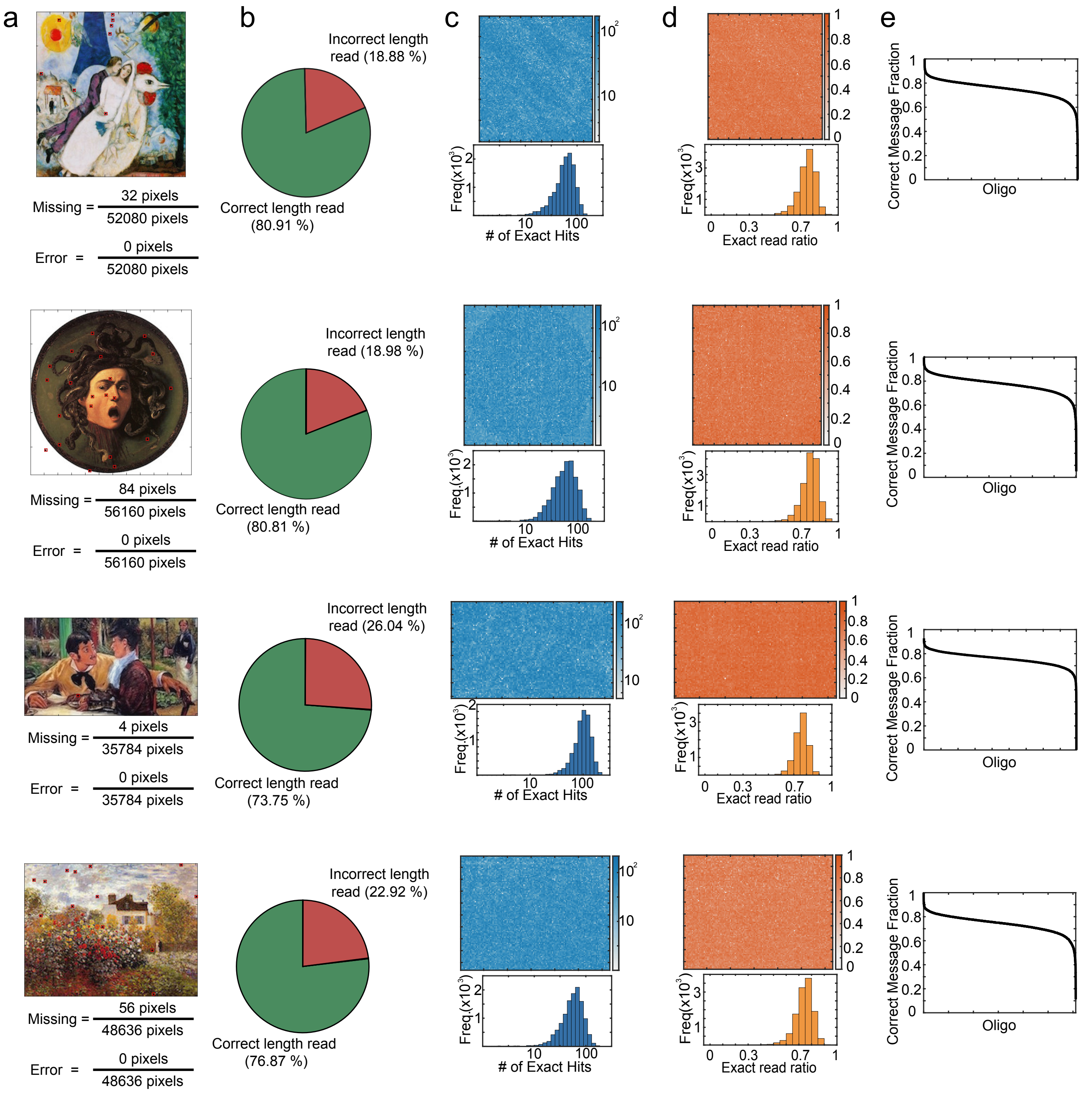

Supplementary Figure. 9: NGS analysis of amplified subpools for Images 1 through 4, using the first Twist pool. **(a)** Visualr epresentation of pixels; missing pixel blocks are boxed in red. **(b)** Fraction of NGS reads with correct vs. incorrect lengths for the address and message. All reads with incorrect lengths were discarded in subsequent analysis. **(c)** Distribution o fNGS reads for each pixel block corresponding to the exact expected sequence. **(d)** Fraction of NGS reads at each pix elcorresponding to the exact expected sequence. **(e)** Distribution of fraction of NGS reads with exact expected sequence acro ssall oligos.

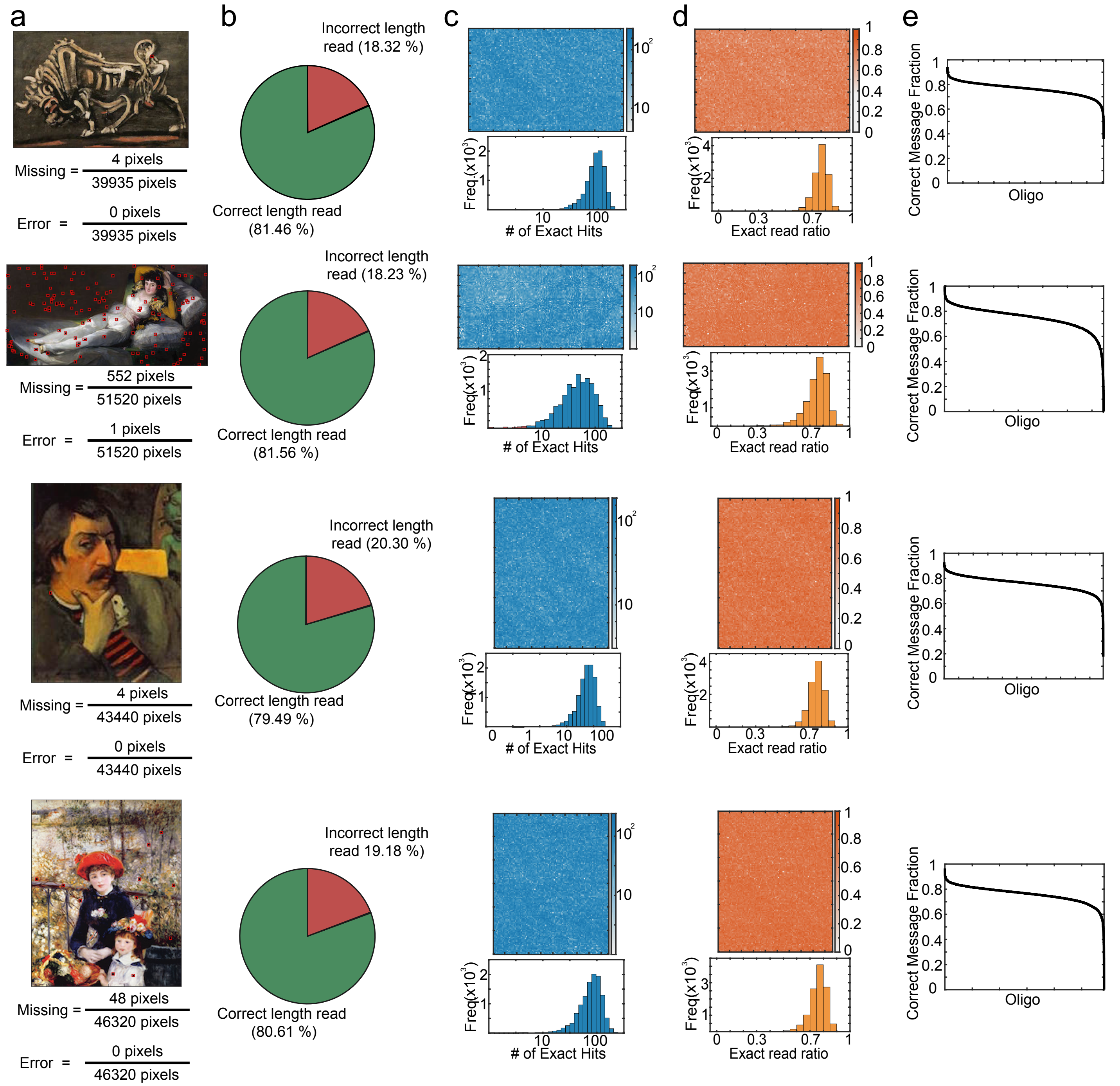

Supplementary Figure. 10: NGS analysis of amplified subpools for Images 5 through 8, using the first Twist pool. **(a)** Visual representation of pixels; missing pixel blocks are boxed in red. **(b)** Fraction of NGS reads with correct vs. incorrec tlengths for the address and message. All reads with incorrect lengths were discarded in subsequent analysis. **(c)** Distributio nof NGS reads for each pixel block corresponding to the exact expected sequence. **(d)** Fraction of NGS reads at each pix elcorresponding to the exact expected sequence. **(e)** Distribution of fraction of NGS reads with exact expected sequence acro ssall oligos.

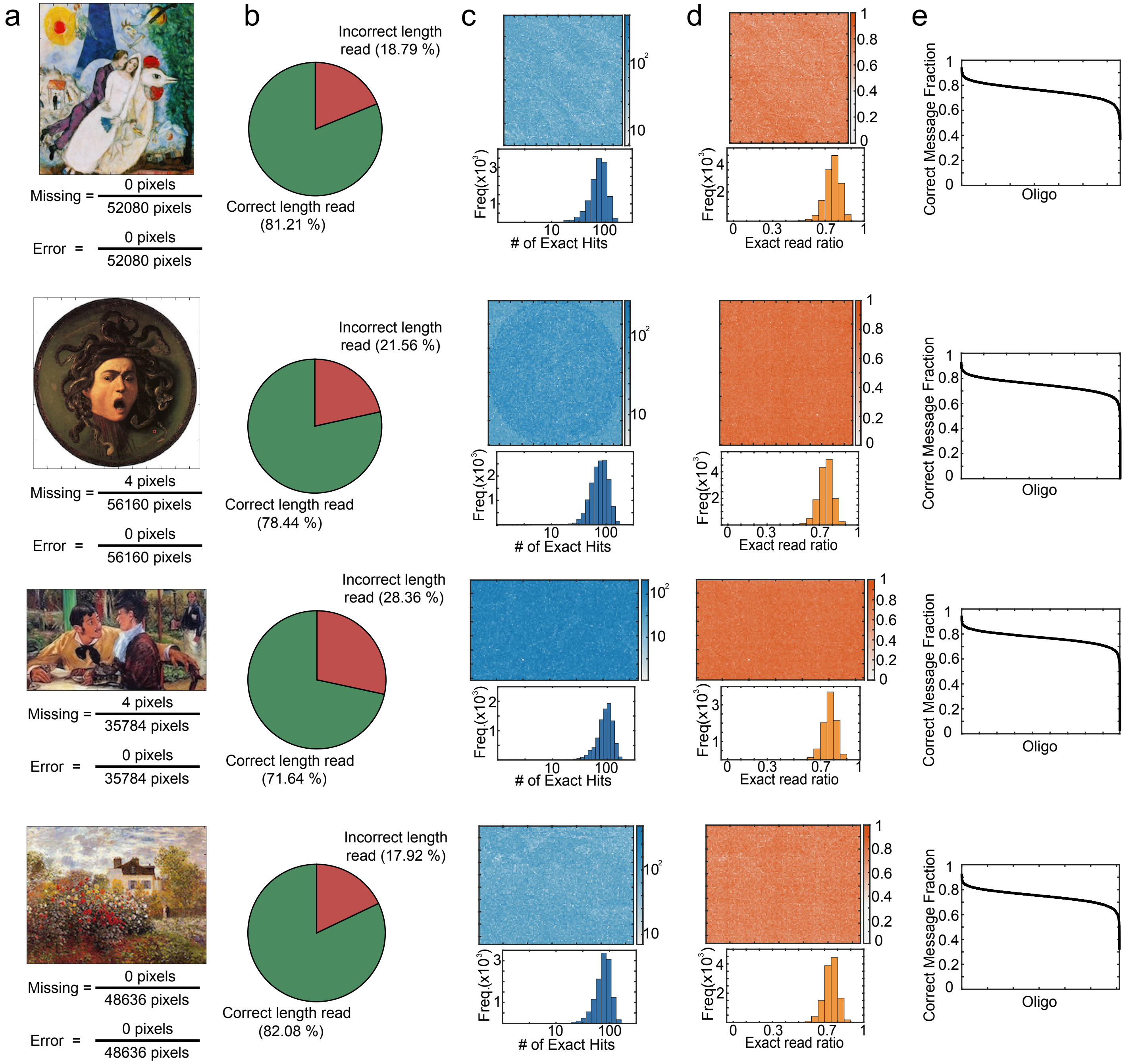

Supplementary Figure. 11: NGS analysis of amplified subpools for Images 1 through 4, using the combined first Twist poola nd second repair Twist pool. **(a)** Visual representation of pixels; missing pixel blocks are boxed in red. **(b)** Fraction of NGS reads with correct vs. incorrect lengths for the address and message. All reads with incorrect lengths were discarded in subsequent analysis. **(c)** Distribution of NGS reads for each pixel block corresponding to the exact expected sequence. **(d)** Fraction of NGS reads at each pixel corresponding to the exact expected sequence. **(e)** Distribution of fraction of NGS reads with exact expected sequence across all oligos.

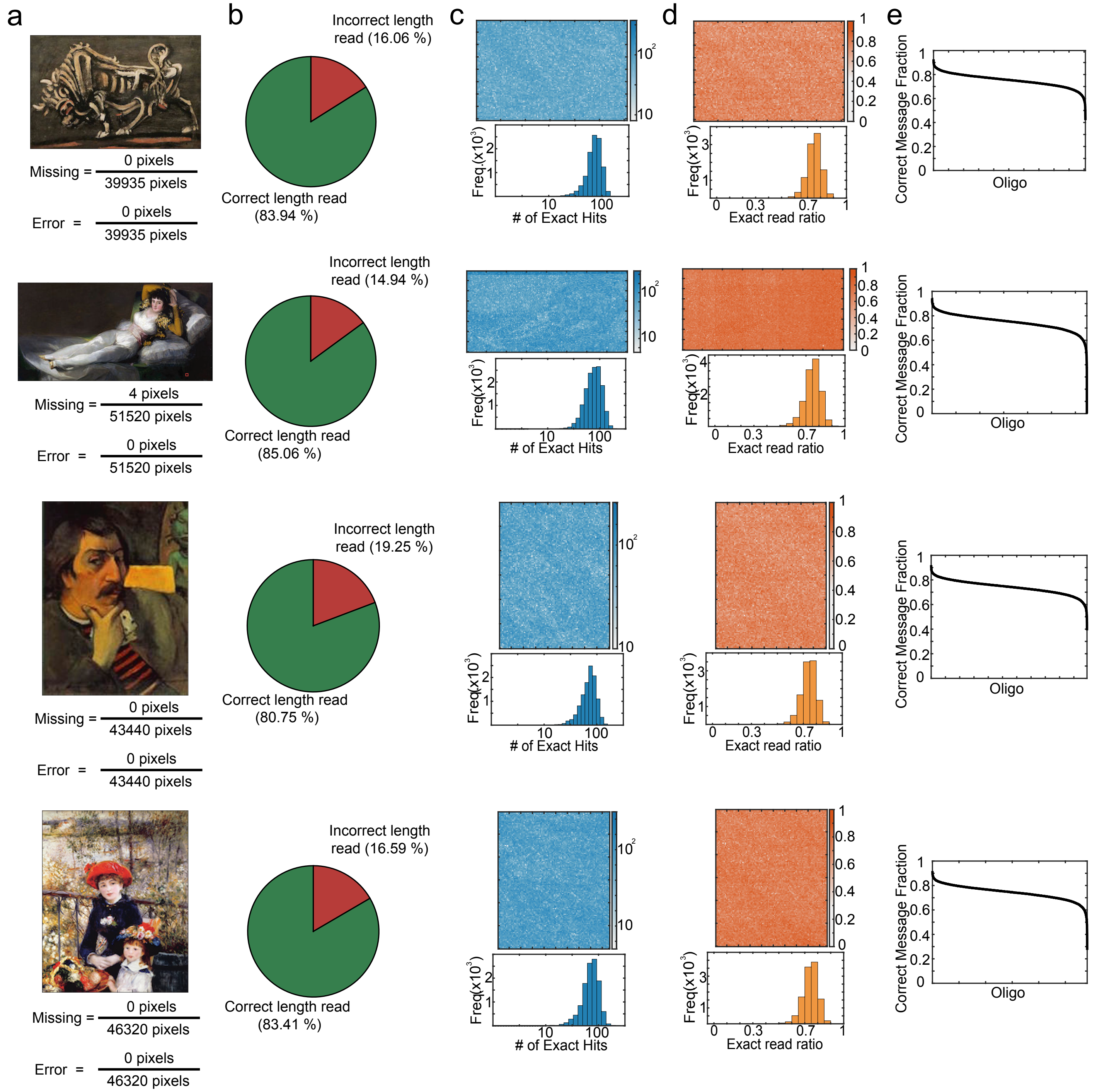

Supplementary Figure. 12: NGS analysis of amplified subpools for Images 5 through 8, using the combined first Twist poola nd second repair Twist pool. **(a)** Visual representation of pixels; missing pixel blocks are boxed in red. **(b)** Fraction of NGS reads with correct vs. incorrect lengths for the address and message. All reads with incorrect lengths were discarded in subsequent analysis. **(c)** Distribution of NGS reads for each pixel block corresponding to the exact expected sequence. **(d)** Fraction of NGS reads at each pixel corresponding to the exact expected sequence. **(e)** Distribution of fraction of NGS reads with exact expected sequence across all oligos.

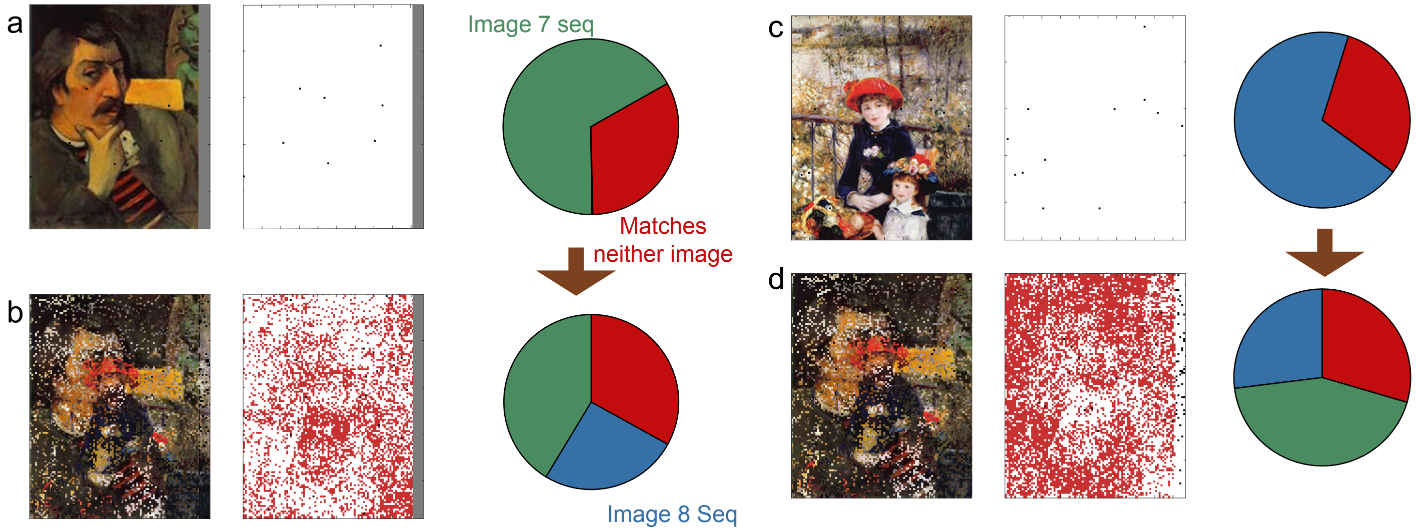

Supplementary Figure. 13: NGS analysis of information solutions comprising mixing images 7 and 8. **(a)** Informations olution stored for 1 week, initially prepared with image 7 bound to truth marker and Image 8 bound to false marker. Lefts hows a visual representation of the consensus NGS read for each pixel. Middle shows the pixels that correspond to thee xpected Image 7 message (white), the Image 8 message (red), or is missing (black). Right shows the distribution of NGS reads that match Image 7, Image 8, or neither. **(b)** Information solution in (1) after erasing by incubating 5 minutes at 95 °C. **(c)** Information solution stored for 1 week, initially prepared with image 8 bound to truth marker and Image 7 bound to false marker. **(d)** Information solution in (4) after erasing by incubating 5 minutes at 95 °C.

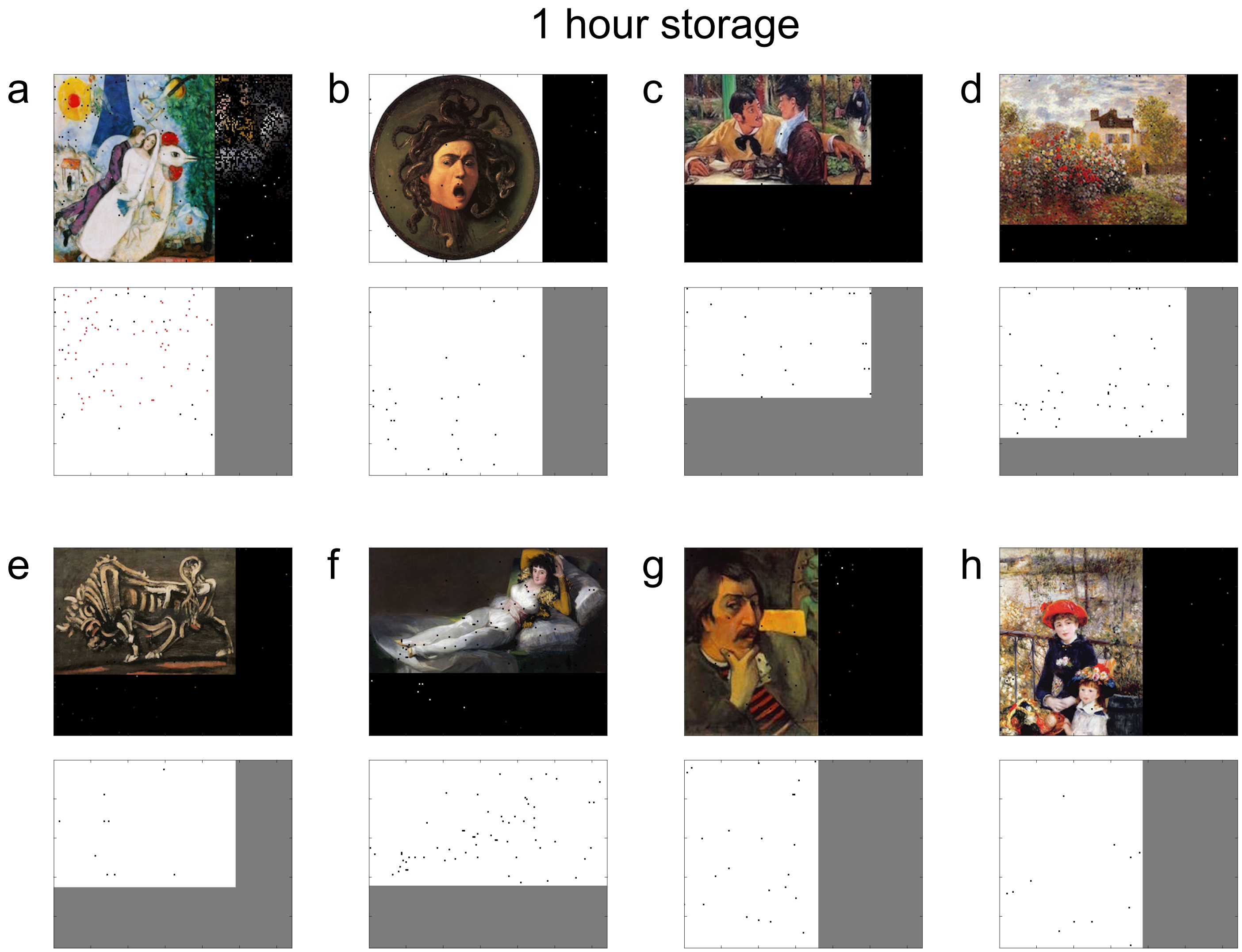

Supplementary Figure. 14: NGS analysis of information solutions comprising mixing images 1 through 8. The informations olution stored for 1 hour at 25 °C. Panels **(a)** through **(f)** correspond to information solutions in which image 1 through 8 were pre-hybridized to the truth marker, respectively. All seven other images were pre-hybridized to the false marker.

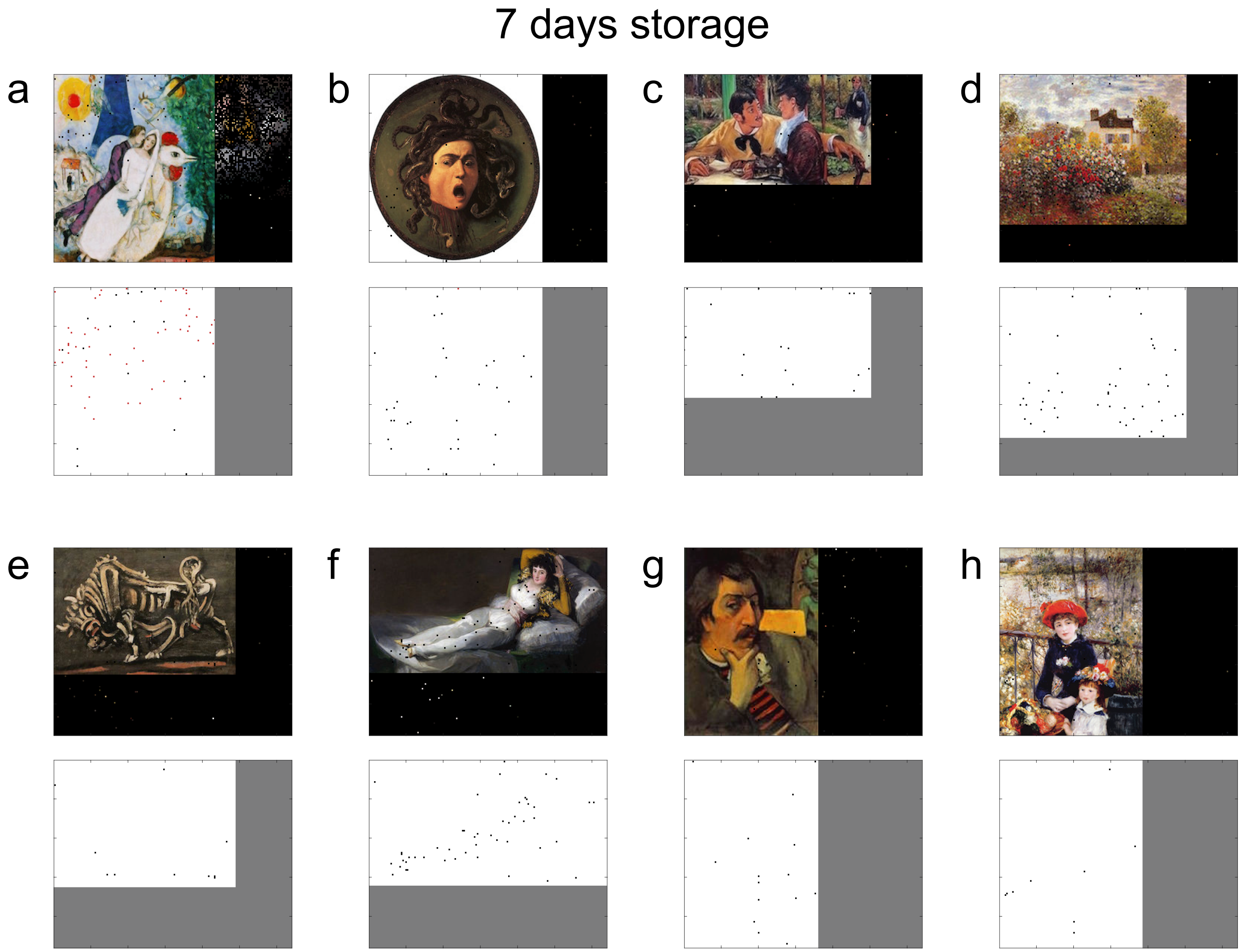

Supplementary Figure. 15: NGS analysis of information solutions comprising mixing images 1 through 8. The informations olution stored for 7 days at 25 °C. Panels **(a)** through **(f)** correspond to information solutions in which image 1 through 8 were pre-hybridized to the truth marker, respectively. All seven other images were pre-hybridized to the false marker.

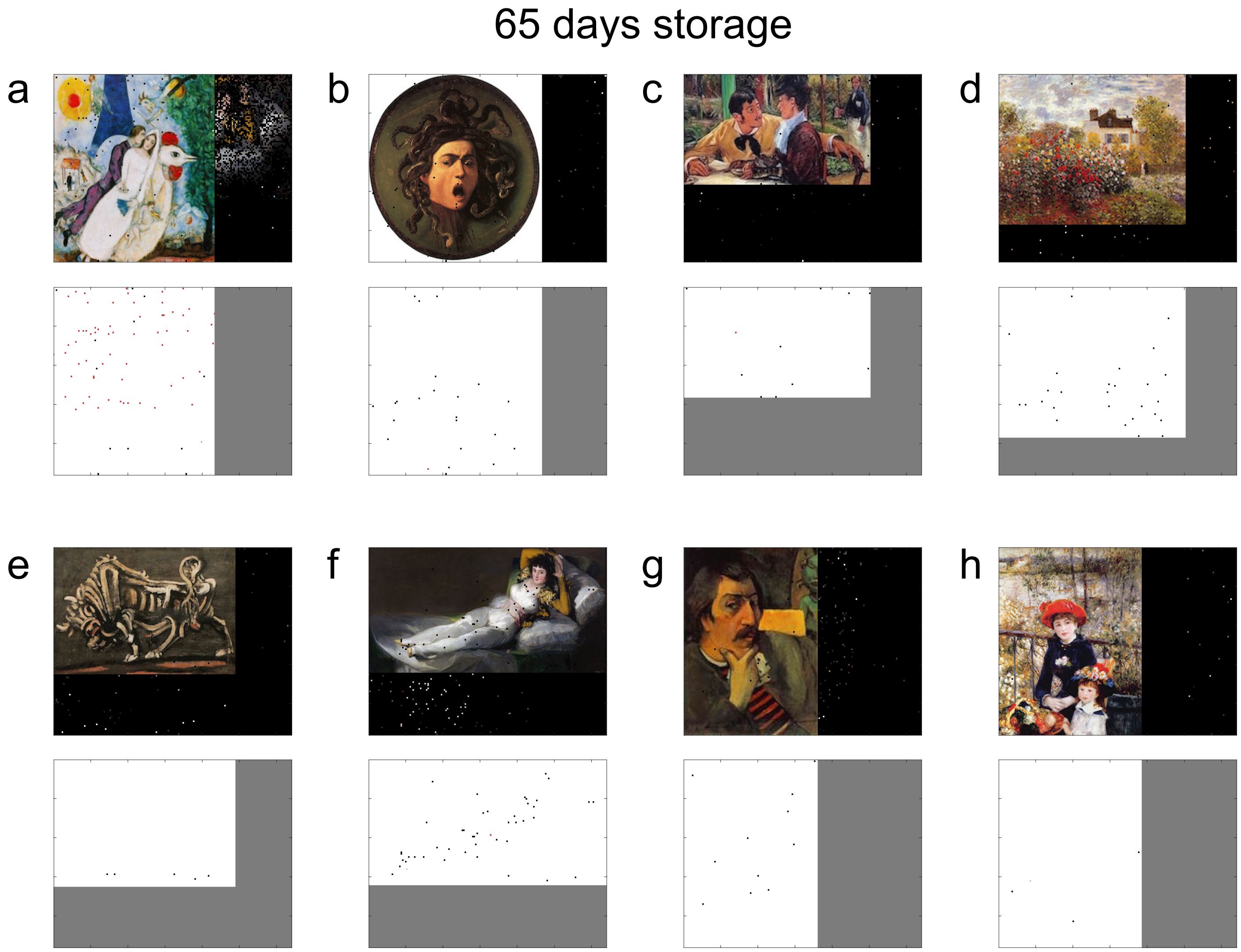

Supplementary Figure. 16: NGS analysis of information solutions comprising mixing images 1 through 8. The informations olution stored for 65 days at 25 °C. Panels **(a)** through **(f)** correspond to information solutions in which image 1 through 8 were pre-hybridized to the truth marker, respectively. All seven other images were pre-hybridized to the false marker.

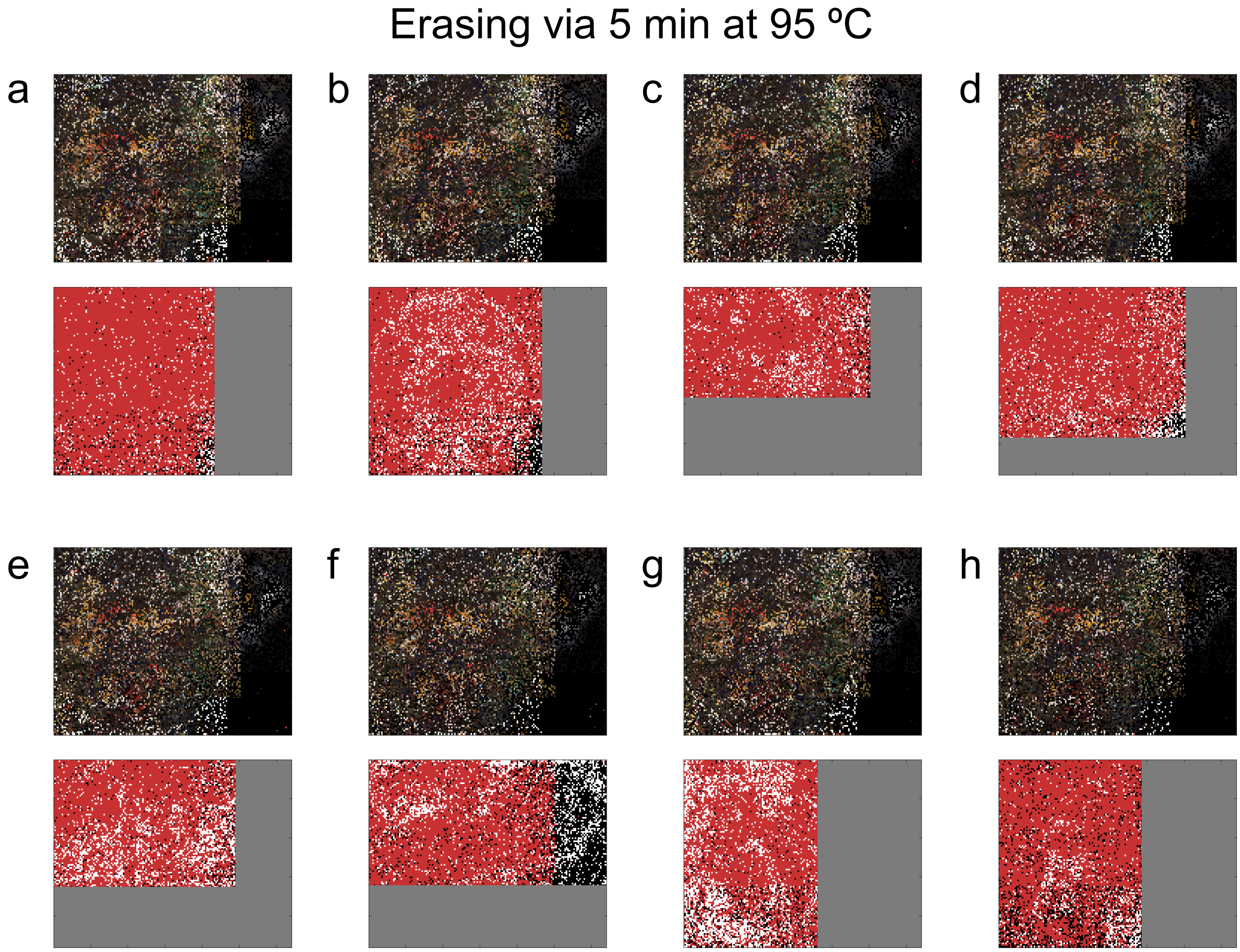

Supplementary Figure. 17: NGS analysis of information solutions comprising mixing images 1 through 8. The informations olution stored for 1 hour at 25 °C, and then erased by heating to 95 °C for 5 min. Panels **(a)** through **(f)** correspond to information solutions in which image 1 through 8 were pre-hybridized to the truth marker, respectively. All seven other images were pre-hybridized to the false marker.

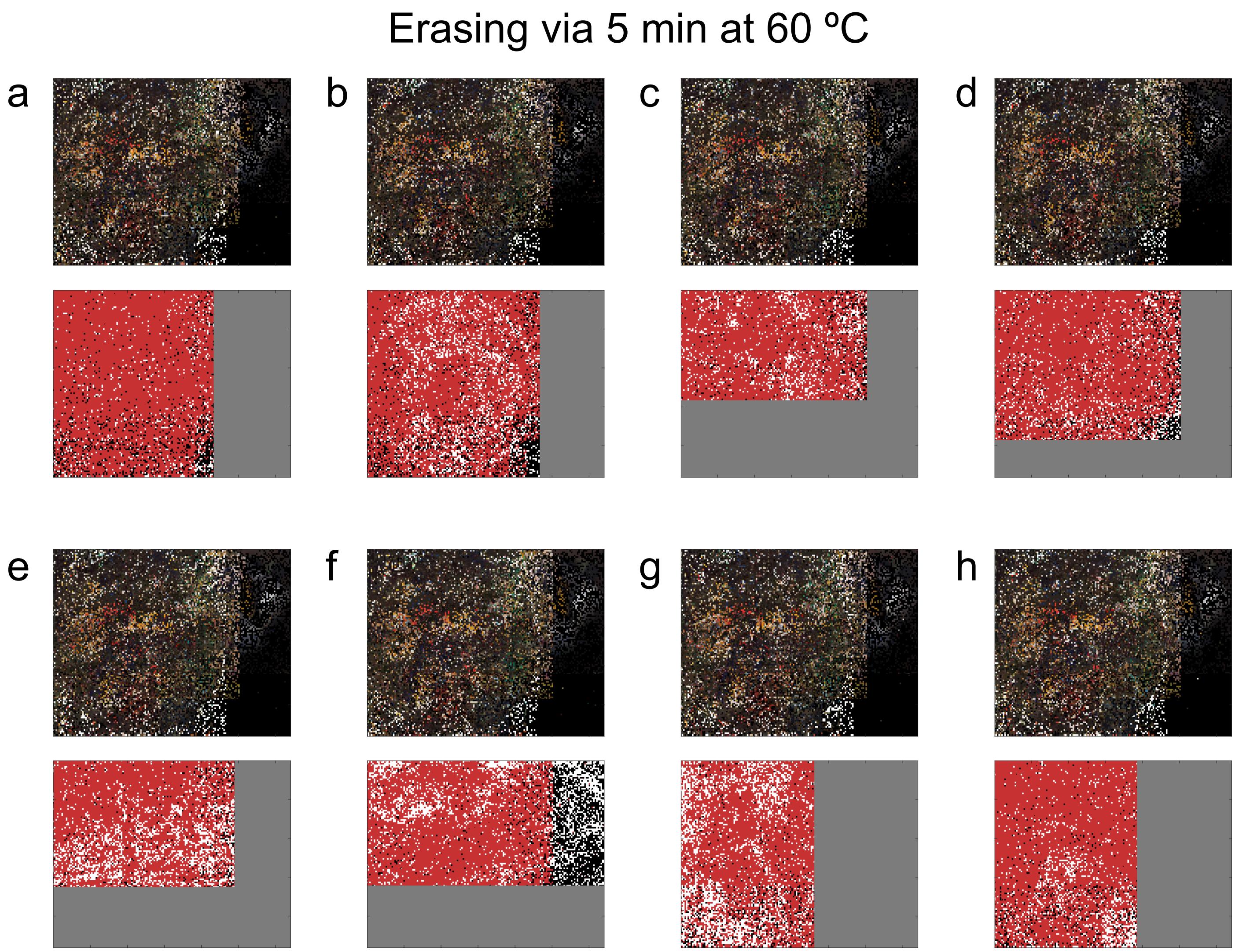

Supplementary Figure. 18: NGS analysis of information solutions comprising mixing images 1 through 8. The informations olution stored for 1 hour at 25 °C, and then erased by heating to 60 °C for 5 min. Panels **(a)** through **(f)** correspond to information solutions in which image 1 through 8 were pre-hybridized to the truth marker, respectively. All seven other images were pre-hybridized to the false marker.

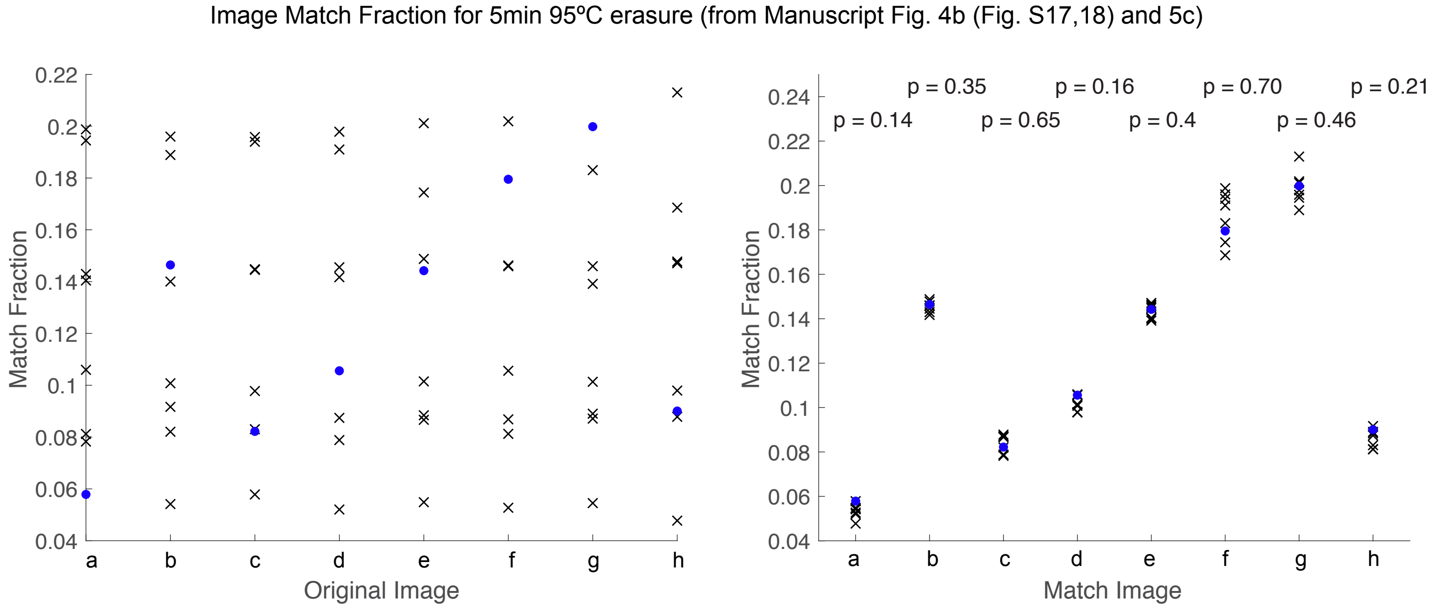

Supplementary Figure. 19: Fraction of pixel blocks matched to each Match Image, based on the Original (intended) image. Each reported p-value is calculated based on a one-way t-test with 7 degrees of freedom.

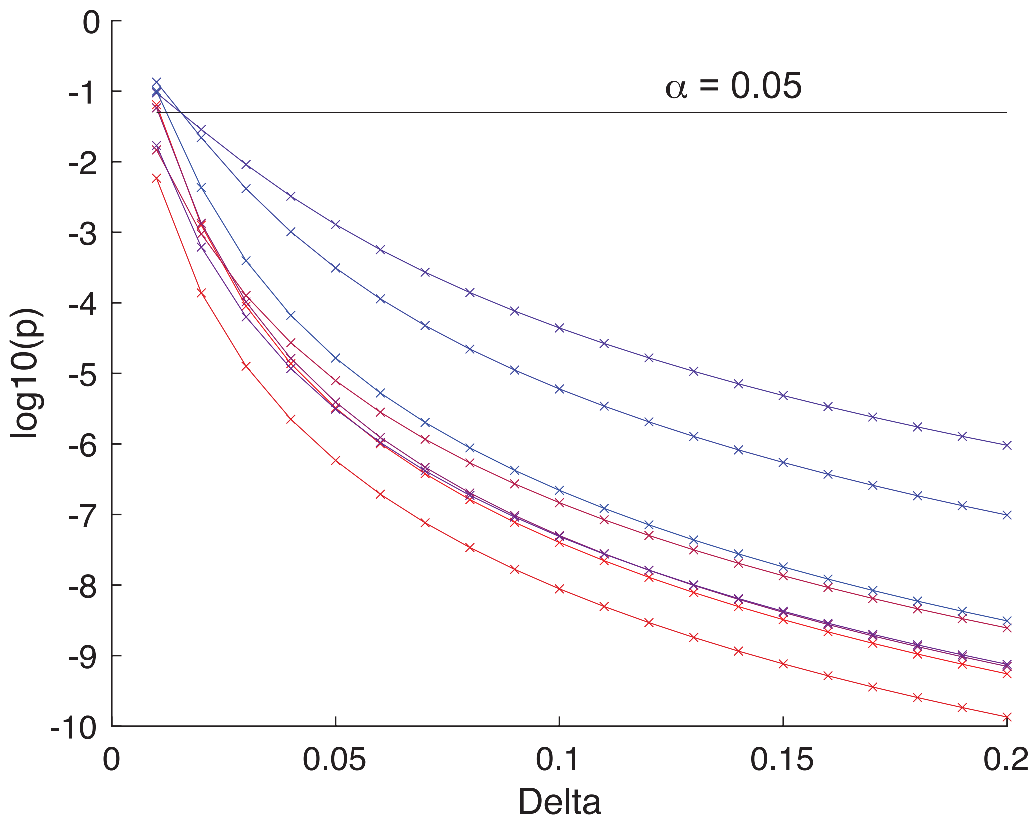

Supplementary Figure. 20: Computed p-values for evaluating whether more than Delta fraction of the information remainsi n the erased images, based on analysis of the matching matrix in Table S1.

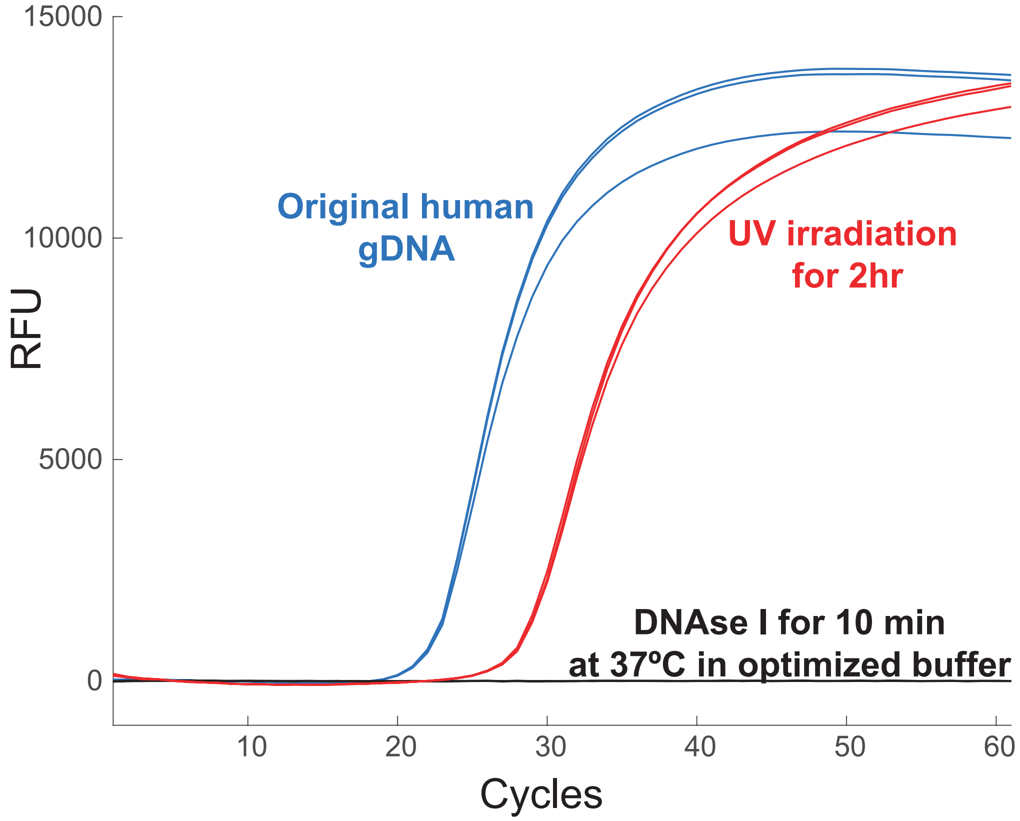

Supplementary Figure. 21: qPCR results on original 100ng human gDNA sample (blue), UV irradiated DNA sample (red), and DNAse I-treated sample (black). There is roughly a 6 cycle Ct delay between the blue and red traces, indicating that roughly 98% of the original DNA was rendered unamplifiable through UV irradiation. There was no observed amplification after DNAse I treatment, indicating that over 99.99% of the original DNA was rendered unamplifiable by DNAse I.

Supplementary Table

	Match Im1	Match Im2	Match Im3	Match Im4	Match Im5	Match Im6	Match Im7	Match Im8
Orig. Im1	0.0579	0.1430	0.0783	0.1060	0.1404	0.1988	0.1945	0.0812
Orig. Im2	0.0541	0.1464	0.0821	0.1008	0.1400	0.1960	0.1889	0.0917
Orig. Im3	0.0578	0.1445	0.0822	0.0978	0.1448	0.1940	0.1958	0.0830
Orig. Im4	0.0520	0.1417	0.0788	0.1056	0.1456	0.1910	0.1979	0.0874
Orig. Im5	0.0549	0.1488	0.0867	0.1015	0.1442	0.1744	0.2011	0.0884
Orig. Im6	0.0527	0.1459	0.0868	0.1056	0.1463	0.1795	0.2019	0.0812
Orig. Im7	0.0545	0.1460	0.0871	0.1013	0.1392	0.1830	0.1998	0.0890
Orig. Im8	0.0477	0.1477	0.0878	0.0980	0.1471	0.1686	0.2130	0.0900

Supplementary Table. S1: Fraction of pixel blocks matched to each Match Image, based on the Original (intended) image.
