# Supplementary figures and images for: Metastable hybridization-based DNA information storage to allow rapid and permanent erasure
Source: Nat Commun. 2020 Oct 6;11:5008. doi: 10.1038/s41467-020-18842-6 (PMC7538566; doi:10.1038/s41467-020-18842-6)

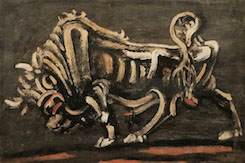

Supplement: Supplementary file 4 — Supplementary Software [file 41467_2020_18842_MOESM4_ESM.zip › Code/For Demo/Example Data for Eecode/Original Image.bmp]

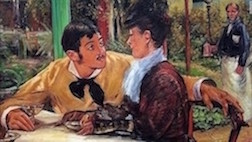

Supplement: Supplementary file 4 — Supplementary Software [file 41467_2020_18842_MOESM4_ESM.zip › Code/Original Images/3.bmp]

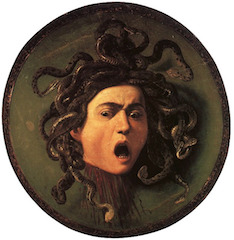

Supplement: Supplementary file 4 — Supplementary Software [file 41467_2020_18842_MOESM4_ESM.zip › Code/Original Images/2.bmp]

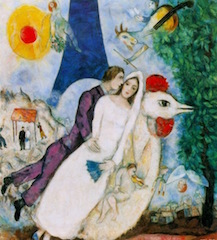

Supplement: Supplementary file 4 — Supplementary Software [file 41467_2020_18842_MOESM4_ESM.zip › Code/Original Images/1.bmp]

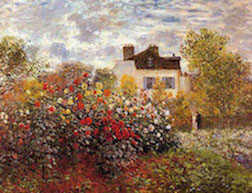

Supplement: Supplementary file 4 — Supplementary Software [file 41467_2020_18842_MOESM4_ESM.zip › Code/Original Images/4.bmp]

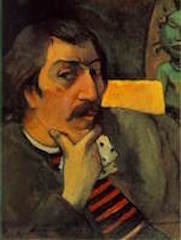

Supplement: Supplementary file 4 — Supplementary Software [file 41467_2020_18842_MOESM4_ESM.zip › Code/Original Images/6.bmp]

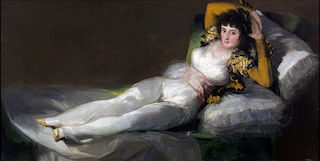

Supplement: Supplementary file 4 — Supplementary Software [file 41467_2020_18842_MOESM4_ESM.zip › Code/Original Images/7.bmp]

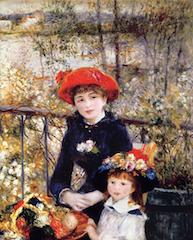

Supplement: Supplementary file 4 — Supplementary Software [file 41467_2020_18842_MOESM4_ESM.zip › Code/Original Images/8.bmp]
