# Supplementary material for: Metastable hybridization-based DNA information storage to allow rapid and permanent erasure
Source: Nat Commun. 2020 Oct 6;11:5008. doi: 10.1038/s41467-020-18842-6 (PMC7538566; doi:10.1038/s41467-020-18842-6)
Supplement: Supplementary file 5 — Description of Additional Supplementary Files [file 41467_2020_18842_MOESM5_ESM.pdf]

Title: Supplementary Data 1
Description: Excel document detailing Twist oligo pool and primers sequences

Title: Supplementary Software
Description: Zip file containing software/code information
